# Sarcoidosis-Lymphoma Syndrome Presenting As Bony Vertebral Metastasis: A Case Report and Literature Review

**DOI:** 10.7759/cureus.13227

**Published:** 2021-02-08

**Authors:** Long Di, Christopher P Wang, Joseph Tang, Robert Macaulay, Nam Tran

**Affiliations:** 1 Neurological Surgery, University of South Florida Morsani College of Medicine, Tampa, USA; 2 Pathology, Moffitt Cancer Center, Tampa, USA; 3 Neurological Surgery, Moffitt Cancer Center, Tampa, USA

**Keywords:** sarcoidosis, lymphoma, vertebral metastasis, sarcoidosis-lymphoma syndrome, lumbar, cervical, thoracic, kyphoplasty, neurosurgery, neurological surgery

## Abstract

Sarcoidosis preceding a diagnosis of lymphoma has been a reported phenomenon termed sarcoidosis-lymphoma syndrome. Skeletal metastasis is extremely rare. Here, we detail a case of sarcoidosis-lymphoma syndrome presenting as a lumbar vertebral metastasis with suspected associated intracranial lesions. A 72-year-old man with a history of follicular lymphoma presented with symptomatic central nervous system (CNS) lesions with concurrent lumbar vertebral metastases visualized with CT and MRI. Rituximab, cyclophosphamide, doxorubicin hydrochloride, vincristine sulfate, and prednisone (R-CHOP) with dexamethasone treatment resulted in significant radiographic regression of his intracranial lesions with dramatic symptomatic improvement. Out of concern for compression fracture of his lytic lumbar lesions, kyphoplasty with biopsy was performed showing lymphocytes that were positive for cluster-of-differentiation 10 (CD10), CD20, and B-cell lymphoma 2 (Bcl2). The patient was diagnosed with CNS and vertebral sarcoidosis-lymphoma syndrome and began treatment with high-dose methotrexate. Including the present case, only four occurrences of sarcoidosis-lymphoma syndrome with bony involvement have been described. We detail our own experience and summarize all previous literature. While rare, sarcoidosis-lymphoma may present with CNS and lytic bone involvement; in these cases, symptomatic severity, as well as an effective response to steroid treatment, underscore the importance of an accurate and prompt diagnosis.

## Introduction

Sarcoidosis-lymphoma syndrome describes the rare phenomena in which a diagnosis of lymphoma is made in the setting of chronic sarcoidosis [1]. Incidence of sarcoidosis is uncommon, occurring at a rate between five to 40 cases per 100,000 people per year [2], and the involvement of lymphoma is sparse. Granulomatous reactions associated with sarcoidosis are commonly seen in the same organ as the lymphoma but can also be found in other hematopoietic organs [3]. Approximately one to five percent of patients with sarcoidosis die from sarcoidosis related complications [4], while mortality for lymphoma is significantly higher [5].

It is extremely rare for the diagnosis of lymphoma to precede sarcoidosis, as well as to include skeletal involvement. To date, there have only been three reports published in the literature where patients have presented with bony metastasis of sarcoidosis-lymphoma syndrome with only one study showing vertebral involvement. We present a case in which L5 biopsy revealed noncaseating granulomatous cells in the vertebral body following the identification of central nervous system (CNS) and spinal lesions with imaging, with a history of lymphoma.

Our patient presented with initial focal bulbar neurologic deficit and headache prior to L5 biopsy which resolved with treatment involving Dexamethasone, Keppra, and Fioricet. Lesion size regressed significantly since initial encounter. One-month post-L5 biopsy and kyphoplasty, the patient returned with worsened neurological condition and for consultation. Scattered non-caseating granulomas with multinucleated giant cells were found from the biopsy and the patient was started on high dose methotrexate.

## Case presentation

**Figure 1:**
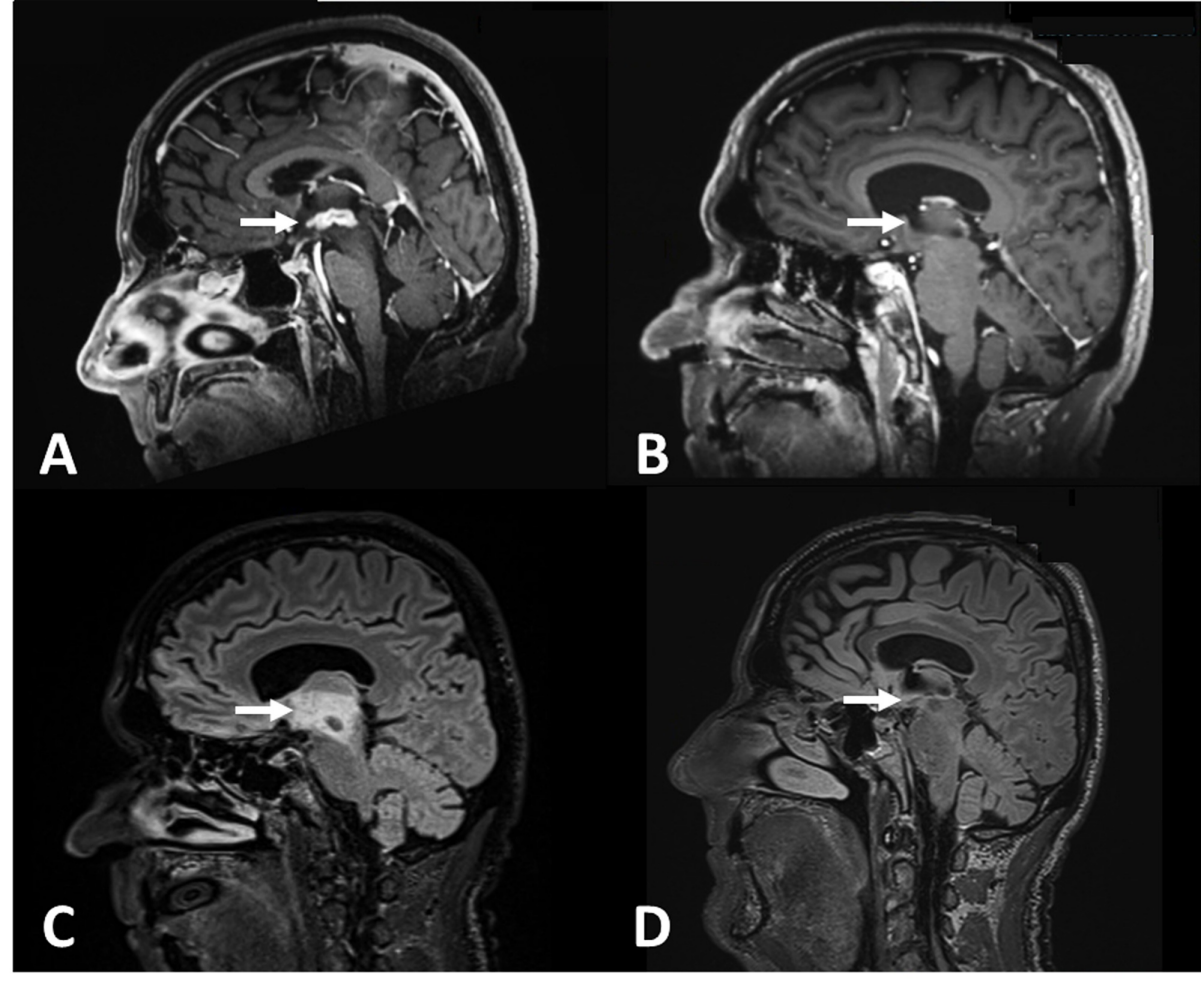
Sagittal MPRAGE MRI post administration of gadolinium contrast agent and T2 FLAIR sequences before and after the patient completed R-CHOP with dexamethasone treatment. A: Sagittal magnetization-prepared rapid acquisition with gradient echo (MPRAGE) MRI shows presence of a large, contrast-enhancing third ventricular lesion. B. Two-week follow up MRI after completion of R-CHOP and steroid treatment, there is a significant reduction in lesion size. C/D: T2 fluid-attenuated inversion recovery (FLAIR) sequences show a similar degree of reduction in the volume of FLAIR hyperintensity.

A 72-year-old man with a history of basal cell carcinoma, squamous cell carcinoma, and low-grade follicular lymphoma was referred to our clinic for evaluation of diplopia, headache, tongue paralysis, dysarthria, and CNS lesions. Approximately two months prior to presentation, the patient developed progressively worsening double vision and headache while driving warranting emergency department admission. Head CT revealed cerebral edema and concomitant MRI demonstrated multiple ring-enhancing, intracranial lesions in the third ventricle and thalamus as well as lumbar spinal lesions consistent with metastatic disease. Abdominal and thoracic CT also showed diffuse lymphadenopathy in mediastinal, paraaortic, retroperitoneal, and axillary lymph nodes. Left axillary node biopsy showed low-grade follicular lymphoma that was positive for cluster-of-differentiation 10 (CD10), CD20, and B-cell lymphoma 2 (Bcl2) as well as fluorescence in situ hybridization (FISH) positive for t(14;18). Dexamethasone, Keppra, and Fioricet were initiated with symptom improvement and the patient was referred to our clinic for further evaluation. At the time of admission, the patient’s neurologic symptoms had resolved, and steroids were discontinued. Repeat MRI showed significant regression in lesion size rendering them too small to safely biopsy. The patient was discharged without an official diagnosis for his CNS and spinal lesions but began rituximab, cyclophosphamide, doxorubicin hydrochloride, vincristine sulfate, and prednisone (R-CHOP) for his lymphoma (Figure 1).

**Figure 2:**
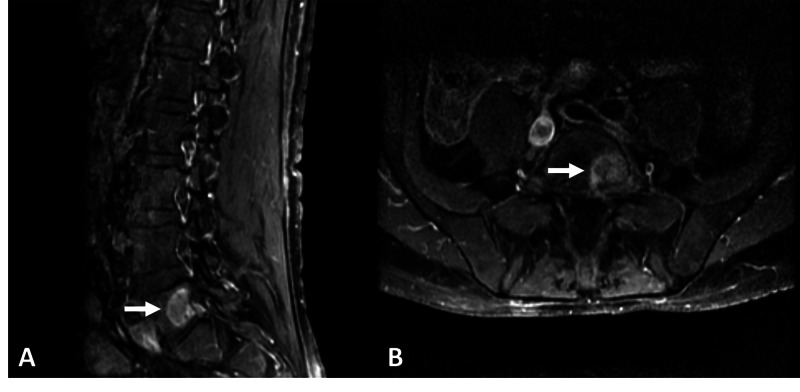
Lumbar spinal metastasis. Sagittal (A) and axial (B) T1 post-contrast enhancement MRI of a large ring enhancing lesion invading the left, posterolateral aspect of the L5 vertebral body

One month later, he returned for a consultation on biopsy of his spinal lesion (Fig. 2). Given the lytic character of the lesion and the high risk of compression fracture, a plan for L5 kyphoplasty with biopsy was made. After obtaining informed consent, the patient was taken to the operating room. Biplanar fluoroscopy was performed with the patient in prone position to localize the thoraco-lumbar levels. Incisions were made lateral to the vertebral bodies and Jamshidi needles were used to cannulate the pedicles with fluoroscopic navigation. Upon reaching the L5 pedicle vertebral junction, biopsy samples were obtained and sent for permanent tissue analysis. Semisolid methyl methacrylate was then injected into the vertebral bodies. Final lateral fluoroscopic projections were conducted to confirm adequate cement placement prior to closure. The patient was discharged without complications.

**Figure 3:**
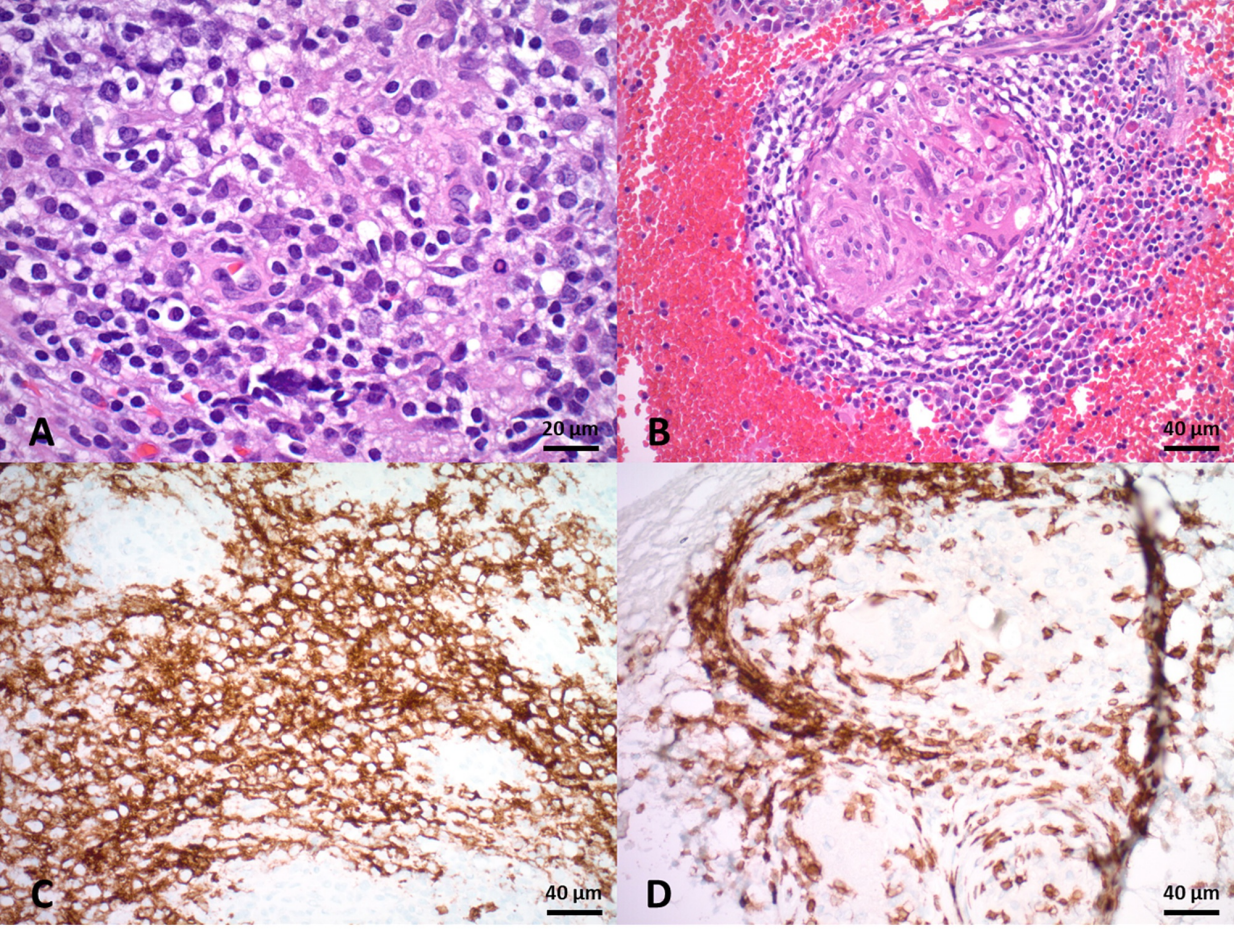
Histological analysis of the L5 lesion biopsy. A: Sections showing patchy lymphomatous infiltration consisting of medium-to-large sized cells with scant eosinophilic cytoplasm and oval hyperchromatic nuclei with coarse chromatin with variable numbers of eosinophils. B: Scattered noncaseating granulomas are present in a background of lymphomatous infiltrate. C: Lymphocytic infiltrate stains positive for CD20. D: Lymphoid cells also stain positive for CD3 and surround a granuloma.

Upon completion of a cycle of R-CHOP and cessation of steroids, his diplopia and dysarthria again worsened considerably at two-week post-operative follow-up. Biopsy of his L5 lesion returned a diagnosis consistent with diffuse large B-cell lymphoma (DLBCL). Scatter mitoses were present in addition to scattered non-caseating granulomas with multinucleated giant cells (Fig. 3). Given his prior response to dexamethasone, symptomatic deterioration after steroid cessation, and results of his L5 biopsy, a diagnosis of CNS and vertebral lymphoma-sarcoid syndrome was made, and the patient began treatment with high-dose methotrexate. The patient was currently undergoing treatment at the time of manuscript preparation. 

## Discussion

Here, we present a case of a 72-year-old man with CNS and vertebral sarcoidosis-lymphoma syndrome treated with R-CHOP, steroids, and high-dose methotrexate. Sarcoidosis is a chronic, multi-system disease of unknown origin characterized by the presence of non-caseating granulomas [6]. The clinical picture presents on radiographs as mediastinal and hilar lymphadenopathy, sometimes with the involvement of the right paratracheal lymph nodes [7]. Sarcoidosis-lymphoma syndrome, as first described by Brincker, is the occurrence of sarcoidosis preceding a diagnosis of lymphoma [7]. The cause of this dual presentation has been hypothesized to be chronic sarcoidosis causing an altered immune reaction with auto-immune elements, later causing malignant lymphoma [8].

Despite previously reported cases of sarcoidosis-lymphoma metastases invading soft tissue, boney involvement is rare [9, 10]. Our literature review returned only four previously reported cases including the present case (Table 1) [11-13]. The median age at diagnosis was 60 years. In two cases (50%), sarcoidosis was diagnosed first while in the other two cases (50%) lymphoma was diagnosed initially. The median time between initial diagnosis and diagnosis of the secondary pathology was 1.25 months. In three cases (75%), the lymphoma was diagnosed as a diffuse-large B-cell lymphoma while lymphoma pathology was not reported in English in the last case [11]. Metastases presented as a lymphocytic, granulomatous invasion of the vertebral or lower extremity skeleton. In all cases, CHOP or R-CHOP therapy was used to treat the lymphoma yielding a good therapeutic response. The Median follow-up was 12 months.

**Table 1:** Cases of sarcoidosis-lymphoma syndrome with skeletal involvement. TBD = time-before-diagnosis of concurrent sarcoidosis-lymphoma; M = male; F = female; mo = month; DLBCL = diffuse large B-cell lymphoma; R-CHOP = rituximab, cyclophosphamide, doxorubicin hydrochloride, vincristine sulfate, and prednisone; MTX = methotrexate; Gy = gray

Study	Year	Age	Initial Dx	TBD	Sex	Lymphoma Type	Clinical presentation	Treatment	Tumor Location	Histopathology	Outcome	Reccurence?	Last follow up (mo)
Mori et al. [11]	2009	63	Sarcoidosis	1 mo	F	B-cell Lymphoma	Back pain	R-CHOP	Lumbar vertebra	Noncaseating granulomas with CD5+, CD20+ tumor cells showing K-chain monoclonality	Complete response	No	N/A
Kobayashi et al. [12]	2001	57	Lymphoma	1.5 mo	F	DLBCL	Right foot pain and swelling	CHOP	Right talus	Epithelioid granuloma with	Some response	No	12
Marks et al. [13]	2018	54	Sarcoidosis	7 yr	M	DLBCL	Pain and swelling in both ankles	R-CHOP + MTX + local 36 Gy Radiotherapy	Bilateral distal tibiae	Diffuse infiltrates of CD20+ lymhoid cells	Complete response	No	56
Di et al. (current study)	2019	72	Lymphoma	1 mo	M	DLBCL	Diplopia, headache, dysarthria	R-CHOP + Dexamethasone + MTX	C1, L1, L5 vertebrae; right iliac, left inferior sacrum	CD10+, CD20+, Bcl2+, FISH+ for t(14;18)	Complete response	No	2.3

The relationship between sarcoidosis and lymphoma is well-established, supported by a finding demonstrating an increased risk of 14.1% of Hodgkin’s lymphoma in patients with sarcoidosis [14]. Lymphoma also occurred 11 times more frequently in patients with respiratory sarcoidosis than expected compared to the general population in report by Cohen et al [14]. Local sarcoid-like reactions may develop in draining lymph nodes but may occur on top of and in conjunction with systemic sarcoidosis [15]. Granulomatous reactions are most often seen in the same organ as the lymphoma but can be found in other hematopoietic organs [3].

Having a primary manifestation of sarcoidosis-lymphoma syndrome followed by metastasis is exceedingly rare and not well-studied in literature. While sarcoidosis is not a common disease, occurring at a rate between five to 40 cases per 100,000 people per year [2], the involvement of lymphoma is sparse [16]. Multiple imaging and laboratory methodologies should be used to confirm the diagnosis. Common modalities include 18-fluorodeoxyglucose positron emission computed tomography (18 FDG PET-CT) to select appropriate biopsy sites [17], serum protein electrophoresis and blood analysis [18], and X-ray radiography. Testing should be done on a case-by-case basis based on the clinical presentation; in the case of a bony metastasis, gallium (Ga)-67 scintigraphy may be used to localize tumors and inflammation [12].

The approach of treating sarcoidosis-lymphoma syndrome involves therapies that actively treat both diseases. The most frequently used chemotherapy regimen against lymphoma is R-CHOP (cyclophosphamide, doxorubicin, vincristine, prednisone, and rituximab) [17]. In the case of malignant lymphoma of the bone, 40 Gy irradiation was also used in a female patient presenting with lymphoma in the right foot [12]. Sarcoidosis is treated with immunosuppressive drugs or corticosteroids such as prednisone and prednisolone [19].

The outcome of patients with lymphoma associated with sarcoidosis compared to patients with lymphoma alone is still under study. Some studies have indicated improved rates of relapse-free survival in patients experiencing sarcoid-like reactions in conjunction with cancer diseases [20]. However, in the largest retrospective, multicenter series to date, Chalayer et al. did not discover any added survival benefit when lymphoma was associated with sarcoidosis. Given the rarity of disease presentation, additional reports may be needed to arrive at a definitive conclusion. In our own experience and literature review, it seems that treatment with R-CHOP and corticosteroids is an effective therapy with most patients achieving remission and symptom resolution. High-dose methotrexate therapy may be employed as salvage therapy in the setting of symptomatic worsening, as was seen in our patient following completion of R-CHOP and dexamethasone therapy. 

## Conclusions

We present a case of sarcoidosis-lymphoma syndrome presenting with CNS and vertebral involvement with concomitant focal neurologic deficits. We supplement our case with a literature review that includes all previously reported cases of boney sarcoidosis-lymphoma metastasis. Multiple radiographic techniques followed by tissue biopsy may be used to reach a definitive diagnosis. Given the dramatic response to chemotherapy and corticosteroid treatment, prompt diagnosis can expedite the management of symptomatic manifestations and achieve remission.
